# Factors secreted by monosodium urate crystal-stimulated macrophages promote a proinflammatory state in osteoblasts: a potential indirect mechanism of bone erosion in gout

**DOI:** 10.1186/s13075-022-02900-z

**Published:** 2022-09-05

**Authors:** Dorit Naot, Bregina Pool, Ashika Chhana, Ryan Gao, Jacob T. Munro, Jillian Cornish, Nicola Dalbeth

**Affiliations:** 1 Department of Medicine, Faculty of Medical and Health Sciences, University of Auckland, 85 Park Rd, Grafton, Auckland, New Zealand; 2 Department of Surgery, University of Auckland, Auckland, New Zealand

**Keywords:** Gout, Urate, Tophus, Bone, Osteoblast

## Abstract

**Background:**

Tophi are lesions commonly present at sites of bone erosion in gout-affected joints. The tophus comprises a core of monosodium urate (MSU) crystals surrounded by soft tissue that contains macrophages and other immune cells. Previous studies found that MSU crystals directly reduce osteoblast viability and function. The aim of the current study was to determine the indirect, macrophage-mediated effects of MSU crystals on osteoblasts.

**Methods:**

Conditioned medium from the RAW264.7 mouse macrophage cell line cultured with MSU crystals was added to the MC3T3-E1 mouse osteoblastic cell line. Conditioned medium from the THP-1 human monocytic cell line cultured with MSU crystals was added to primary human osteoblasts (HOBs). Matrix mineralization was assessed by von Kossa staining. Gene expression was determined by real-time PCR, and concentrations of secreted factors were determined by enzyme-linked immunosorbent assay.

**Results:**

In MC3T3-E1 cells cultured for 13 days in an osteogenic medium, the expression of the osteoblast marker genes *Col1a1*, *Runx2*, *Sp7*, *Bglap*, *Ibsp*, and *Dmp1* was inhibited by a conditioned medium from MSU crystal-stimulated RAW264.7 macrophages. Mineral staining of MC3T3-E1 cultures on day 21 confirmed the inhibition of osteoblast differentiation. In HOB cultures, the effect of 20 h incubation with a conditioned medium from MSU crystal-stimulated THP-1 monocytes on osteoblast gene expression was less consistent. Expression of the genes encoding cyclooxygenase-2 and IL-6 and secretion of the proinflammatory mediators PGE_2_ and IL-6 were induced in MC3T3-E1 and HOBs incubated with conditioned medium from MSU crystal-stimulated macrophages/monocytes. However, inhibition of cyclooxygenase-2 activity and PGE_2_ secretion from HOBs indicated that this pathway does not play a major role in mediating the indirect effects of MSU crystals in HOBs.

**Conclusions:**

Factors secreted from macrophages stimulated by MSU crystals attenuate osteoblast differentiation and induce the expression and secretion of proinflammatory mediators from osteoblasts. We suggest that bone erosion in joints affected by gout results from a combination of direct and indirect effects of MSU crystals.

**Supplementary Information:**

The online version contains supplementary material available at 10.1186/s13075-022-02900-z.

## Introduction

Bone damage is a frequent consequence of tophaceous gout. The tophus is a well-organized structure consisting of a core of monosodium urate (MSU) crystals surrounded by soft tissue containing innate and adaptive immune cells, particularly macrophages [1]. Bone damage in tophaceous gout results from imbalanced bone remodeling at the bone-tophus interface. Bone erosion at the tophus–bone interface results from excessive bone resorption by osteoclasts that is not adequately balanced by bone formation by osteoblasts, whereas aberrant osteoblast activity may drive new bone formation, resulting in sclerosis and spur formation [2, 3].

Mechanisms that underlie bone damage have been investigated using in vitro models and histological analysis of joint samples from people with gout. A study of osteoblastic cell lines and primary osteoblasts from rat and human found that MSU crystals reduce osteoblast viability and differentiation [4]. Histological analysis of joints affected by bone erosion demonstrated MSU crystals in direct contact with bone cells or separated from bone by a rim of inflammatory tissue [5]. Macrophages are abundant in this tissue and are stimulated by MSU crystals to secrete multiple proinflammatory factors that are likely to affect bone cells [6]. In a previous study, we tested the macrophage-mediated effects of MSU crystals on osteocytes and found that a conditioned medium from MSU-stimulated macrophages promoted a shift in osteocyte function that favored bone resorption and inflammation [5].

The indirect effects of MSU crystals on osteoblasts have not been studied previously. Osteoblast activity is central to generating the characteristic bone phenotype in joints affected by tophaceous gout and is likely to arise from a combination of direct and indirect effects exerted by the tophi. The aim of the current study was to determine the indirect, macrophage-mediated effect of MSU crystals on osteoblast phenotype using two in vitro model systems: the MC3T3-E1 mouse osteoblastic cell line and human primary osteoblasts (HOBs).

## Methods

### Experimental design

Two experimental systems were used in the study. The first used two mouse cell lines: RAW264.7 macrophages and MC3T3-E1 osteoblastic cells, to model osteoblast differentiation in the presence of soluble factors released from MSU crystal-stimulated macrophages. The second system used the human THP-1 monocytic cell line and HOBs to model the indirect effects of MSU crystals on differentiated osteoblasts.

### Preparation of MSU-stimulated conditioned media 

Endotoxin-free MSU crystals were prepared by recrystallization from uric acid as previously described [7]. The RAW264.7 mouse macrophage cell line (ATCC, Manassas, USA) was used to prepare MSU crystal-stimulated conditioned medium for the study of the indirect effects of MSU crystals in the mouse MC3T3-E1 osteoblastic cell line. RAW264.7 cells were seeded in 24-well plates at 1 × 10^6^ cells/well. The following day, the medium was changed to α-MEM containing L-glutamine and nucleosides, supplemented with 2.5% heat-inactivated FBS and 2.5% heat-inactivated newborn calf serum. MSU crystals were added at concentrations of 0.1, 0.3, and 0.5 mg/mL, and control cultures were not supplemented with MSU crystals. The human monocytic cell line THP-1 (ATCC, Manassas, USA) was used to prepare MSU crystal-stimulated conditioned medium for the study of the indirect effects of MSU crystals in human primary osteoblast cultures. THP-1 cells were seeded in 24-well plates at 1.5 × 10^6^ cells/well in RPMI and 10% FBS. The following day, media were refreshed, and MSU crystals were added at concentrations of 0.3 and 0.5 mg/mL. Control cultures were not supplemented with MSU crystals. All conditioned media samples, harvested from RAW264.7 cells after 24 h of incubation and from THP-1 cells after 20 h, were filtered through 0.2-μm filters to remove any residual MSU crystals. The absence of MSU crystals was confirmed by polarizing light microscopy.

### MC3T3-E1 viability

MC3T3-E1 cells were cultured in αMEM with 0.1% BSA for 24 h. The medium was refreshed, and conditioned media from RAW264.7 cells stimulated with 0, 0.1, 0.3, and 0.5 mg/mL MSU crystals were added at 40% of the final volume. To determine cell viability at 24 h and 48 h of culture, cells were incubated with the alamarBlue® reagent (Life Technologies) for 6 h, and fluorescence was measured using a Synergy 2 multidetection microplate reader (BioTek Instruments Inc., Winooski, VT).

### MC3T3-E1 osteoblastic differentiation

MC3T3-E1 cells were seeded in six-well plates at 5 × 10^4^ cells/well in MEM with 10% FBS and 1 mM sodium pyruvate. When cells became confluent, the medium was changed to αMEM with 15% FBS, 50 μg/mL L-ascorbic acid 2-phosphate (AA2P), and 10 mM β-glycerophosphate. Conditioned media from RAW264.7 MSU crystal-stimulated and control cells were added at 40% of the final volume. Samples of cells and media were harvested for gene expression and measurement of secreted factors before the addition of MSU crystal-stimulated media (day 0) and on days 1, 6, and 13. Media were changed twice weekly. Samples collected on days 6 and 13 had media replacement 48 h before harvest. Mineral formation was determined in MC3T3-E1 cells cultured for 21 days and stained for minerals using von Kossa stain.

### Human primary osteoblast cultures

The collection of human bone samples was approved by the New Zealand Ministry of Health and Disability Ethics Committee (HDEC approval no. NTX/05/06/058*).* All study participants provided their written informed consent.

Cultures of primary osteoblasts (HOBs) were prepared from normal human trabecular bone obtained from patients undergoing knee or hip arthroplasty, using a method based on Robey and Termine [8]. For the study of the indirect effect of MSU crystals on HOBs, cells were seeded in 6-well plates at 5 × 10^4^ cells/well in DMEM with 5% FBS and 10 µg/mL AA2P. The following day, media were changed to DMEM with 0.1% BSA and 10 µg/mL AA2P. Media were refreshed on the following day, and conditioned media from MSU crystal-stimulated THP-1 cells and controls were added, at 40% of the final volume. Cells were harvested and media collected prior to the addition of THP-1 conditioned media and following 2 h and 20 h of incubation. For the study of the role of COX-2 activation in the indirect effect of MSU crystals in HOBs, 1 μM of the COX-2-selective inhibitor (SC-236, Sigma-Aldrich) was added to HOBs for 1 h prior to the addition of conditioned medium from THP-1 cells. An equal volume of the SC-236 vehicle, DMSO, was added to the control wells.

### Analysis of gene expression

The RNeasy mini kit (QIAGEN Pty Ltd, Melbourne, VIC, Australia) was used for RNA extraction, and genomic DNA was removed with the RNase-free DNase set (QIAGEN Pty Ltd). Complementary DNA was synthesized with Superscript III (Thermo Fisher Scientific). TaqMan™ Gene Expression Assays (Life Technologies) were used for multiplex real-time PCR, with FAM-labeled assays for target genes and VIC-labeled 18S ribosomal RNA as endogenous control. The 2^−ΔΔCt^ method was used to calculate the relative levels of gene expression.

### Quantification of secreted factors

Concentrations of factors secreted from RAW264.7 cells, MC3T3-E1 cells, THP-1 cells, and HOBs were determined by enzyme­ linked immunosorbent assay (ELISA). Assays for cytokines from mouse and human origin were purchased from R&D Systems, and PGE_2_ ELISA kit was purchased from Cayman Chemical. Analysis was carried out following the manufacturers’ protocols.

### Statistical analysis

Data were analyzed using the GraphPad Prism Software (v 9.2.0, GraphPad Software, San Diego, USA). For all experiments, data were pooled from three to five biological repeats. In experiments using HOBs, cells from a different patient were used in each biological repeat. Data presented on logarithmic scale were transformed to log_10_ before analysis. Data were analyzed using one-way or two-way analysis of variance (ANOVA) with post hoc Dunnett’s or Sidak’s multiple comparison tests.

## Results

### Conditioned medium from MSU crystal-stimulated RAW264.7 macrophages does not affect MC3T3-E1 cell viability

**Fig. 1:**
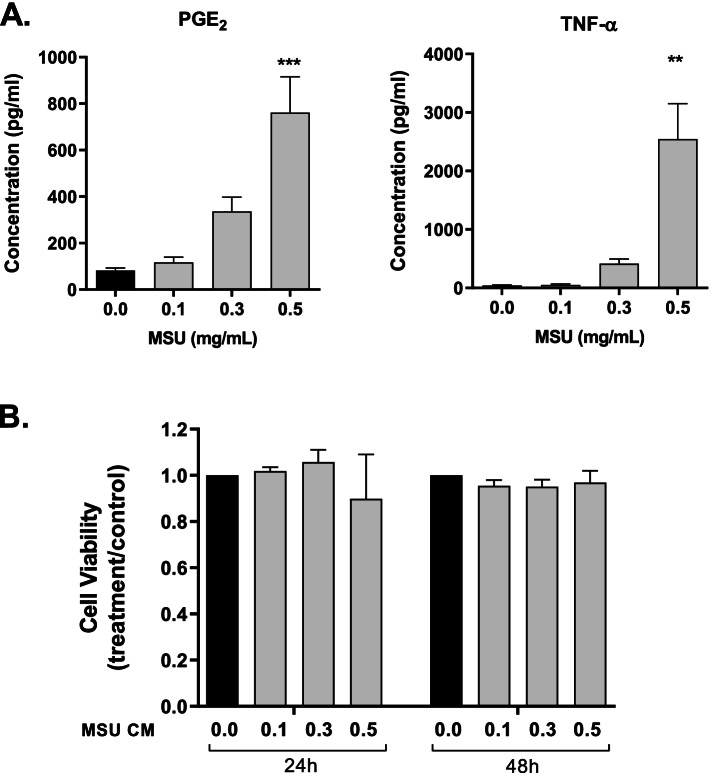
Conditioned medium from MSU crystal-stimulated RAW264.7 macrophages—proinflammatory factors and effect on MC3T3-E1 cell viability. RAW264.7 macrophages were cultured with MSU crystals for 24 h. **A** The concentrations of proinflammatory mediators secreted from MSU crystal-stimulated RAW264.7 cells, measured by ELISA. **B** Conditioned media preparations were added to MC3T3-E1 cells at 40% of the final culture volume. MC3T3-E1 cell viability was determined by the alamarBlue® assay. Means (SEM) of data pooled from three biological repeats are presented. Data were analyzed by one-way ANOVA with post hoc Dunnett’s test. **p < 0.01 and ***p < 0.001 versus control conditioned medium. MSU CM, concentrations of MSU crystals used to prepare conditioned medium from RAW264.7 cells

Conditioned medium for the investigation of the indirect, macrophage-mediated effect of MSU crystals on bone cells was prepared by incubating RAW264.7 macrophages for 24 h with increasing concentrations of MSU crystals. The concentrations of the proinflammatory mediators PGE_2_ and TNF-α increased in the media of RAW264.7 macrophages cultured with MSU crystals (Fig. 1a). In the presence of 0.5 mg/mL MSU crystals, the concentrations of secreted PGE_2_ and TNF-α were approximately 10- and 60-fold higher, respectively, than their concentration in control cultures. IL-1β was present at very low concentrations in RAW264.7 cells conditioned medium and remained low with MSU crystal stimulation (data not shown), and IL-6 was undetected.

To determine the macrophage-mediated, indirect effect of MSU crystals on the viability of osteoblastic cells, conditioned media from RAW264.7 cells incubated with 0, 0.1, 0.3, and 0.5 mg/mL MSU crystals were added to cultures of MC3T3-E1 cells. The conditioned media did not affect MC3T3-E1 viability, as determined by the alamarBlue® assay at 24 h and 48 h of culture (Fig. 1b).

### Conditioned medium from MSU crystal-stimulated RAW264.7 macrophages inhibits osteoblastic differentiation of MC3T3-E1 cells

**Fig. 2:**
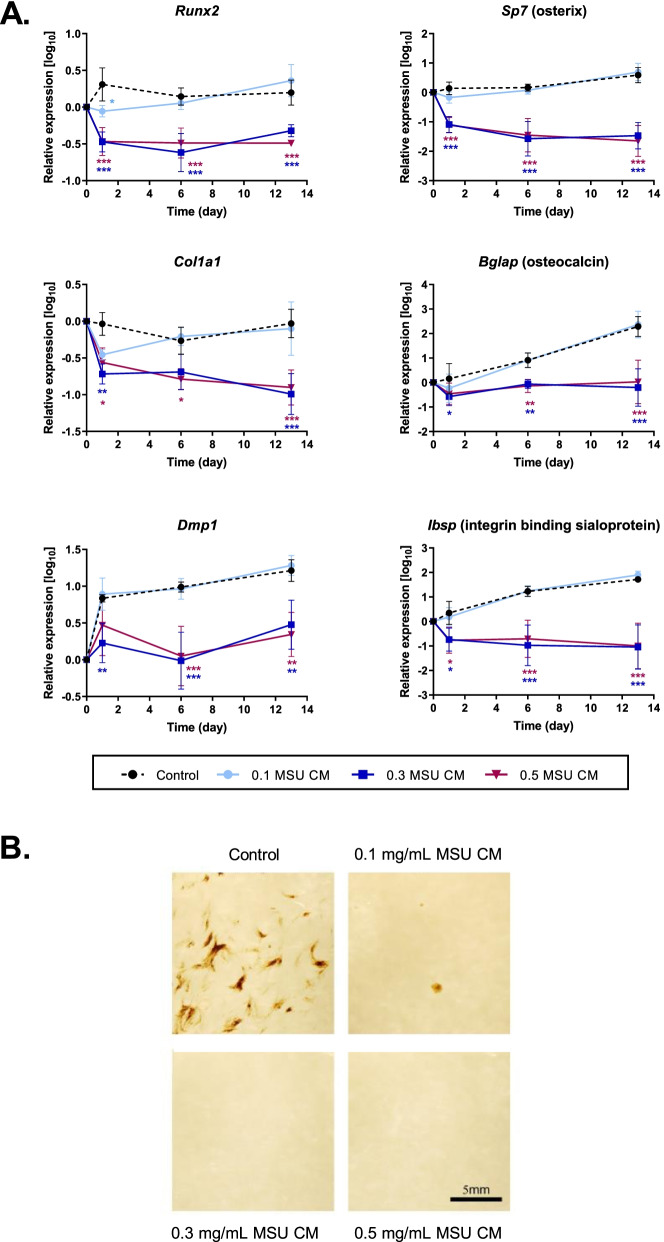
Effects of conditioned medium from MSU crystal-stimulated RAW264.7 macrophages on osteoblastic differentiation of MC3T3-E1 cells. RAW264.7 macrophages were cultured with the indicated concentrations of MSU crystals for 24 h. Conditioned medium from the RAW264.7 cultures was added to MC3T3-E1 cells at 40% of the final volume. **A** Expression of osteoblast marker genes determined by real-time PCR. Means (SEM) of data pooled from three or more biological repeats are presented. Data were analyzed by two-way ANOVA with post hoc Dunnett’s test. *p < 0.05, **p < 0.01, and ***p < 0.001 versus control conditioned medium at that time point. **B** MC3T3-E1 cells were cultured for 3 weeks in an osteogenic medium supplemented with 40% MSU crystal-stimulated conditioned medium from RAW264.7 cells. The representative photomicrographs show mineralized areas (black) and cells (yellow) in cultures stained with von Kossa stain. The experiment was repeated twice with similar results. MSU CM, concentrations of MSU crystals (mg/mL) used to prepare conditioned medium from RAW264.7 cells

Gene expression was determined in MC3T3-E1 cells cultured for 13 days with conditioned media from RAW264.7 cells incubated with 0, 0.1, 0.3, and 0.5 mg/mL MSU crystals (Fig. 2a). The expression of the early osteoblast markers *Runx2*, *Sp7* (osterix), and *Col1a1*, and the late osteoblast markers *Bglap* (osteocalcin), *Dmp1* (dentin matrix acidic phosphoprotein 1), and *Ibsp* (integrin binding sialoprotein) was similar in the control cells, and cells incubated with conditioned medium from RAW264.7 cells stimulated with 0.1 mg/mL MSU crystals. In contrast, conditioned media from RAW264.7 cells incubated with the higher concentrations of MSU crystals inhibited the expression of all six genes. The expression of bone marker genes was already significantly lower in these cultures one day after the addition of the conditioned medium and remained low throughout the 13-day incubation period.

Mineral staining also demonstrated the potent inhibitory effect of the MSU crystal-stimulated conditioned medium on osteogenic differentiation. In MC3T3-E1 cells cultured for 21 days in an osteogenic medium, the formation of a mineral was visible by von Kossa staining in the control cultures, but was greatly reduced in the presence of a conditioned medium from RAW264.7 cells stimulated with 0.1 mg/mL MSU, and blocked in the presence conditioned medium from RAW264.7 cells incubated with higher concentrations of MSU crystals (Fig. 2b).

### Conditioned medium from MSU crystal-stimulated RAW264.7 macrophages inhibits the expression and secretion of OPG, but induces the expression and secretion of proinflammatory mediators from MC3T3-E1 cells

**Fig. 3:**
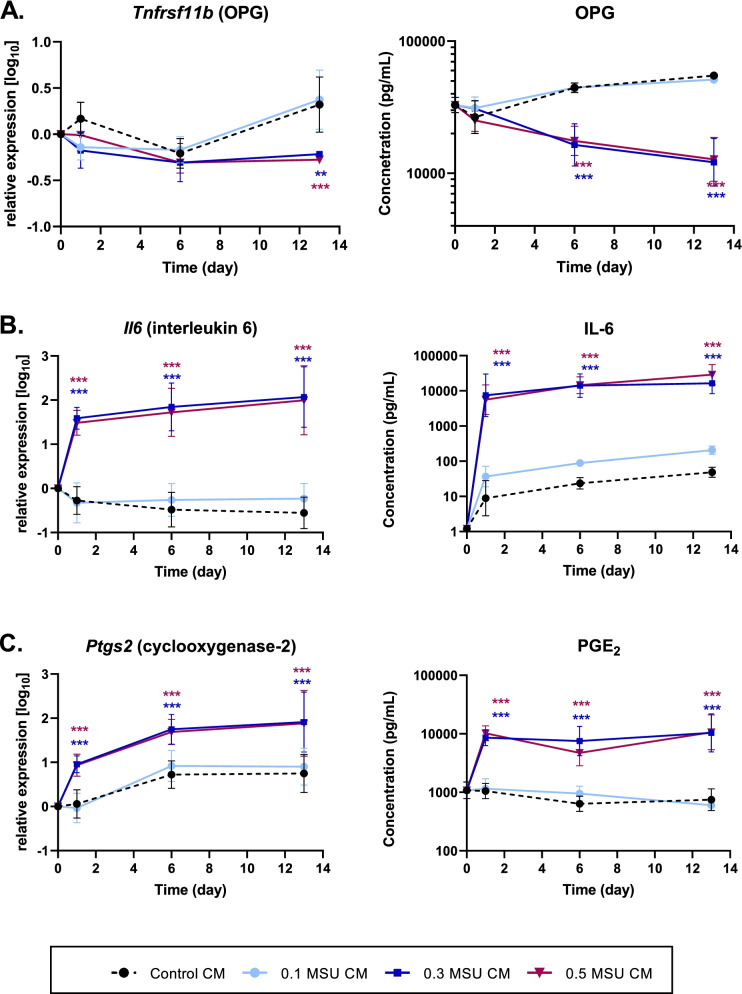
Effects of conditioned medium from MSU crystal-stimulated RAW264.7 macrophages on factor secretion from MC3T3-E1 cells. RAW264.7 macrophages were cultured with the indicated concentrations of MSU crystals for 24 h. Conditioned medium from the RAW264.7 cultures was added to MC3T3-E1 cells at 40% of the final volume. The graphs on the left present gene expression determined by real-time PCR; the graphs on the right present concentrations of secreted factors determined by ELISA. **A** Gene expression and secretion of OPG. **B** Gene expression and secretion of IL-6. **C** Gene expression of *Ptgs2*, encoding the COX-2 enzyme, and secretion of PGE_2_. Means (SEM) of data pooled from three or more biological repeats are presented. Data were analyzed by two-way ANOVA with post hoc Dunnett’s test. *p < 0.05, **p < 0.01, and ***p < 0.001 versus control conditioned medium at that time point; MSU CM, concentrations of MSU crystals used to prepare conditioned medium from RAW264.7 cells

The expression of *Tnfrsf11b* (OPG) was similar in all MC3T3-E1 cultures until day 6, but on day 13 was approximately eight-fold lower in MC3T3-E1 cultures incubated with conditioned media from 0.3 and 0.5 mg/mL MSU crystal-stimulated RAW264.7 cells (Fig. 3a). The concentrations of OPG protein secreted from MC3T3-E1 were also significantly lower in these cultures, with approximately twofold and threefold lower OPG concentrations on days 6 and 13, respectively, in cultures incubated with conditioned media from 0.3 and 0.5 mg/mL MSU crystal-stimulated RAW264.7 cells. The expression of *Tnfsf11* (RANKL) was undetected in MC3T3-E1 cells. The expression of the *Il6* gene and the secretion of IL-6 were over 600-fold higher in MC3T3-E1 cells cultured with a conditioned medium from RAW264.7 cells stimulated with 0.3 or 0.5 mg/mL MSU crystals (Fig. 3b). The expression of *Ptgs2* (encoding cyclooxygenase-2, COX-2) and the secretion of the end product of the COX-2 pathway, prostaglandin-E2 (PGE_2_), were over 20-fold higher in MC3T3-E1 incubated with these conditioned media preparations (Fig. 3c). The expression of the two genes and secretion of the proinflammatory mediators increased rapidly and remained high throughout the experiment, up to day 13.

### Conditioned medium from MSU crystal-stimulated THP-1 monocytes has a modest effect on the expression of osteoblast marker genes but increases the expression of proinflammatory mediators in human primary osteoblasts

**Fig. 4:**
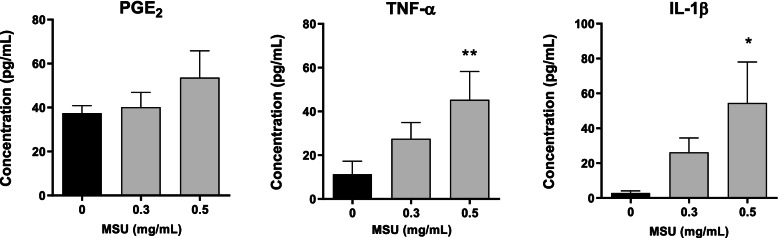
Secretion of proinflammatory mediators from THP-1 monocytes stimulated with MSU crystals. THP-1 monocytes were cultured with MSU crystals for 20 h for preparation of MSU crystal-stimulated and control conditioned media. Concentrations of proinflammatory mediators were determined by ELISA. Means (SEM) of data pooled from three biological repeats are presented. Data were analyzed by one-way ANOVA with post hoc Dunnett’s test. *p < 0.05 and **p < 0.01, versus control without MSU crystals

THP-1, a monocytic cell line derived from human peripheral blood, was used to produce MSU crystal-stimulated conditioned media for the study of the indirect effects of MSU crystals on HOBs. Analysis of THP-1 medium after 20 h of incubation found that 0.5 mg/mL MSU crystals increased the concentrations of the secreted proinflammatory mediators IL-1β and TNF-α, whereas PGE_2_ concentration was similar to that of the control culture (Fig. 4). IL-6 was undetected in conditioned medium from these cultures.

**Fig. 5:**
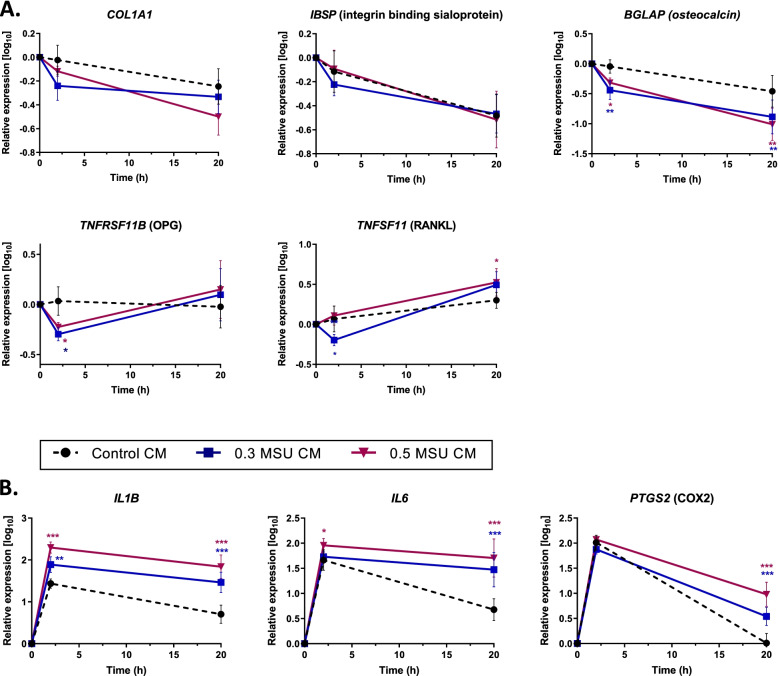
Effects of conditioned medium from MSU crystal-stimulated THP-1 monocytes on gene expression in HOBs. THP-1 monocytes were cultured with MSU crystals for 20 h. Conditioned medium from THP-1 cultures was added to HOB cells at 40% of the final volume. Expression of osteoblast marker genes (**A**) and genes encoding proinflammatory mediators (**B**) was determined by real-time PCR. Means (SEM) of data pooled from three or more biological repeats are presented. Data were analyzed by two-way ANOVA with post hoc Dunnett’s test. *p < 0.05, **p < 0.01, and ***p < 0.001 versus control conditioned medium at that time point. MSU CM, concentrations of MSU [mg/mL] used to prepare conditioned medium from THP-1 cells

Gene expression was determined at 0, 2, and 20 h in HOBs cultured with conditioned medium from MSU crystal-stimulated THP-1 monocytes. The expression of *COL1A1* and *IBSP* was similar in HOBs incubated with MSU-stimulated conditioned medium and control (Fig. 5a). *BGLAP* (osteocalcin) expression was approximately two- and threefold lower in cells incubated with MSU-stimulated conditioned medium at 2 and 20 h, respectively. Modest inhibitory effects of MSU crystal-stimulated conditioned medium were determined at the 2 h time point for the genes encoding OPG and RANKL, but at 20 h RANKL expression was slightly higher in cells incubated with 0.5 mg/mL MSU conditioned medium than in the control.

The expression of the genes *IL1B*, *IL6*, and *PTGS2* increased rapidly in HOBs after the addition of the conditioned medium and peaked at the 2 h time point (Fig. 5b). At 20 h, the expression of *IL1B*, *IL6*, and *PTGS2* declined in the control cultures and remained significantly higher in cultures with MSU crystal-stimulated conditioned medium. In the presence of a conditioned medium from cells stimulated with 0.5 mg/mL MSU, the expression of *IL1B* and *IL6* was approximately 20-fold higher than the controls, and *PTGS2* was approximately tenfold higher than the control.

**Fig. 6:**
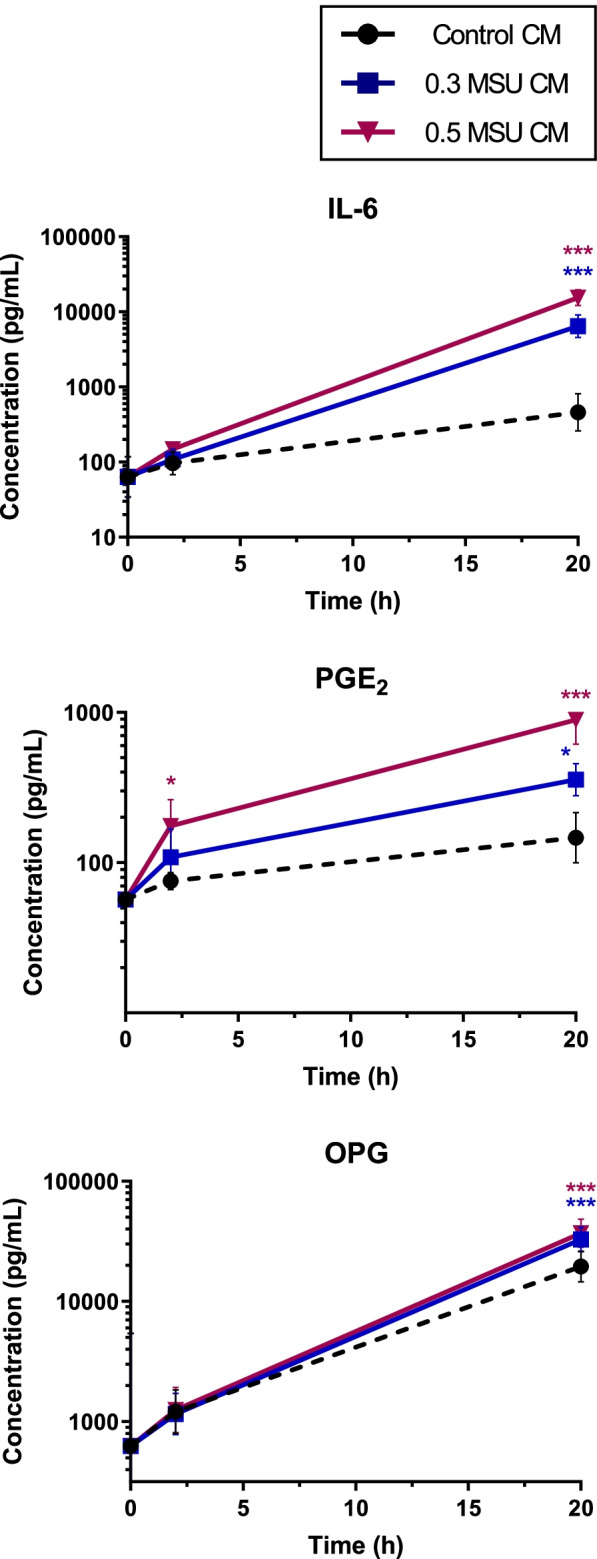
Effects of conditioned medium from MSU crystal-stimulated THP-1 monocytes on factor secretion from HOBs. THP-1 monocytes were cultured with MSU crystals for 20 h. Conditioned medium from THP-1 cultures was added to HOB cells at 40% of the final volume. The concentrations of IL-6, PGE_2_, and OPG secreted from HOBs were measured by ELISA. Means (SEM) of data pooled from three or more biological repeats are presented. Data were analyzed by two-way ANOVA with post hoc Dunnett’s test. *p < 0.05, **p < 0.01, and ***p < 0.001 versus control conditioned medium at that time point. MSU CM, concentrations of MSU [mg/mL] used to prepare conditioned medium from THP-1 cells

The concentrations of IL-6, PGE_2_, and OPG secreted from HOBs increased in response to the addition of MSU crystal-stimulated THP-1 conditioned medium (Fig. 6). At 20 h, the concentrations of IL-6, PGE_2_, and OPG were approximately 25-, 6-, and twofold higher, respectively, in medium harvested from HOBs incubated with 0.5 mg/mL MSU crystal-stimulated conditioned medium than in the controls. Very low concentrations of IL-1β and TNF-α were detected—likely originating from the THP-1 conditioned medium (Fig. S1, Additional File 1).

### COX-2 activation does not play a major role in mediating the response of HOBs to conditioned medium from MSU crystal-stimulated THP-1 cells

**Fig. 7:**
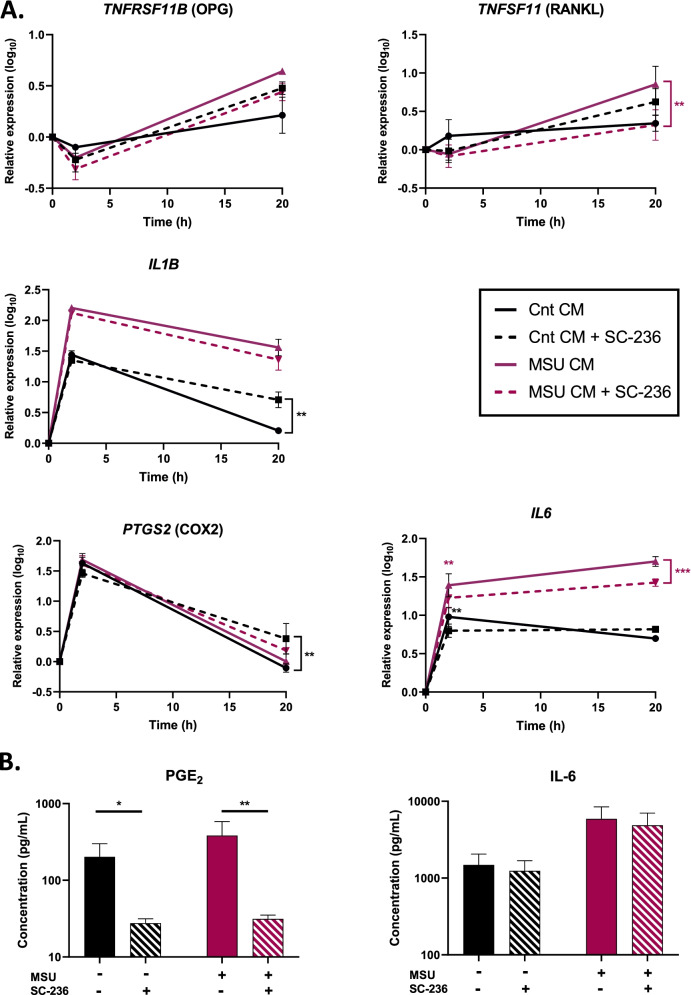
Effects of COX-2 inhibition on HOB response to conditioned medium from MSU crystal-stimulated THP-1 monocytes. Conditioned medium was collected from THP-1 monocytes cultured with or without 0.5 mg/mL MSU crystals for 20 h. The COX-2-selective inhibitor SC-236 was added to HOB cells for 1 h prior to the addition of conditioned medium at 40% of the final volume. **A** Gene expression analyzed by real-time PCR. **B** Secretion of PGE_2_ and IL-6 from HOBs. Data shown are pooled from three biological repeats and are presented as mean (SEM). Data were analyzed by two-way ANOVA with post hoc Sidak’s test. **p < 0.01 and ***p < 0.001 versus control conditioned medium at that time point. Cnt CM and MSU CM, conditioned medium from THP-1 cells incubated without MSU and with 0.5 mg/mL MSU, respectively

In a previous study, we found that inhibition of COX-2 suppressed the inflammatory response induced in MLO-Y4 osteocytic cells by a conditioned medium from MSU crystal-stimulated RAW264.7 macrophages [5]. Here, we have shown that conditioned medium from MSU crystal-stimulated THP-1 cells induced the expression of the gene encoding COX-2 and the secretion of PGE_2_ from HOBs. This suggests a potential role for PGE_2_ as an autocrine mediator of HOBs indirect response to MSU crystals. To test this possibility, the selective inhibitor of COX-2, SC-236, was added to HOB cultures 1 h prior to the addition of conditioned medium from MSU crystal-stimulated THP-1 cells. The increases in gene expression of *TNFSF11* (RANKL) and *IL6* by conditioned medium from MSU crystal-stimulated THP-1 cells seen at the 20 h time point were attenuated by COX-2 inhibition, whereas the expression of *TNFRSF11B* (OPG), *IL1B*, and *PTGS2* (COX-2) did not change with COX-2 inhibition (Fig. 7a). SC-236 inhibited the secretion of PGE_2_ from HOBs incubated with either MSU crystal-stimulated conditioned medium or control conditioned medium (Fig. 7b). However, COX-2 inhibition did not affect the secretion of IL-6 from HOBs.

## Discussion

The study shows that soluble mediators secreted from macrophages in response to stimulation by MSU crystals affect gene expression and secretion of factors from osteoblastic cells. An experimental system based on mouse cell lines was used to model osteoblast differentiation in the presence of soluble factors released from MSU crystal-stimulated macrophages. Conditioned medium from MSU-stimulated RAW264.7 macrophages potently inhibited the osteoblastic differentiation of MC3T3-E1 cells as demonstrated by the reduced expression of osteoblast marker genes and inhibition of matrix mineralization. A second system, based on the human THP-1 monocytic cell line and primary HOBs, was used to model the effects of MSU crystals within the tophus on differentiated osteoblasts. Inhibition of osteoblast marker genes was less consistent in this system. In both experimental systems, conditioned medium from MSU stimulated macrophages/ monocytes increased the expression and secretion of proinflammatory factors from the osteoblastic cells.

For the preparation of the conditioned medium used in our study, macrophage/ monocytic cell lines were incubated with MSU crystals alone, without additional stimulators of inflammation. Activation of the NLRP3 inflammasome and the subsequent release of IL-1β are critical steps in the initiation of the acute inflammatory response to MSU crystals during a gout flare [9, 10]. For this reason, when constructing in vitro systems that model gout flares, MSU crystals are added to macrophages primed with either lipopolysaccharide or phorbol 12-myristate 13-acetate [11, 12]. However, in the current study, we aimed to model the extracellular environment of the osteoblast in tophaceous gout rather than during acute flares. Tophi are not acutely inflamed, and studies mostly find persistent, low-grade inflammation in asymptomatic tophaceous gout [9, 13]. Therefore, we believe that our experimental system represents a relevant in vitro model to examine the indirect, macrophage-mediated effects of MSU crystals on osteoblasts in tophaceous gout.

Conditioned medium from RAW264.7 cells stimulated with 0.3 or 0.5 mg/mL MSU crystals inhibited the expression of *Runx2*, *Sp7*, and *Col1a* and blocked the characteristic increase in the expression of the late osteogenic markers *Dmp1*, *Bglap*, and *Ibsp* in MC3T3-E1 cells. The inhibitory effect of the conditioned medium was also demonstrated in assays of matrix mineralization by MC3T3-E1 cells. The inhibition of matrix mineralization was generally consistent with the gene expression profile, although a conditioned medium generated in RAW264.7 cells with 0.1 mg/mL MSU, which did not affect gene expression, effectively inhibited matrix mineralization. It is possible that under these culture conditions, additional factors required for matrix mineralization, which were not measured in this study, were not produced adequately. A previous study investigating the direct effect of MSU crystals on MC3T3-E1 cells found a similar inhibition of osteoblast marker genes and matrix mineralization [4]. In the current study, we also determined the expression of *COL1A1*, *IBSP*, and *BGLAP* in HOBs following a short incubation with a conditioned medium from MSU crystal-stimulated THP-1 monocytes. In this experimental system, only *BGLAP* expression was lower in the presence of MSU crystal-stimulated conditioned medium than in the control. The direct effect of MSU crystals on HOBs was studied by Bouchard et al. [14]. Analysis of proteins secreted from HOBs in response to 0.3 mg/mL MSU crystals found that alkaline phosphatase and osteocalcin concentrations were reduced to 72% and 58%, respectively, of their concentrations in control cultures. Taken together, these results indicate that in joints affected by tophaceous gout, MSU crystals negatively affect osteoblast function through a combination of direct and indirect mechanisms.

We studied the indirect effects of MSU crystals on OPG and RANKL, two proteins expressed in cells of the osteoblast lineage, which are key regulators of osteoclast formation and bone resorption. The binding of RANKL to RANK, a receptor expressed on osteoclast precursor cells, drives osteoclast differentiation, whereas OPG is a decoy receptor that binds RANKL and inhibits osteoclast differentiation [15]. Studies that determined the circulating concentrations of OPG and RANKL in patients with gout found an imbalance between the two regulator proteins that may be associated with bone erosion in tophaceous gout [16–18]. In our study, incubation with a conditioned medium from MSU crystal-stimulated macrophages/ monocytes reduced OPG gene expression and concentration of secreted protein in long-term cultures of MC3T3-E1 cells, but increased the concentration of secreted OPG in short-term HOB cultures. The different OPG responses in the two culture systems could be related to the nature of the osteoblastic cells used: the first system used a cell line of early osteoblasts induced to differentiate in vitro, and the second used mature osteoblasts obtained from human trabecular bone. Reduced concentration of OPG results in increased bone resorption that could be relevant to bone erosion in gout-affected joints, whereas an increase in OPG concentration could potentially contribute to the formation of the typical boney structures or represent a compensatory mechanism aimed at restoring normal bone metabolism in an environment of excessive bone resorption. Consistent with previous reports, RANKL was not expressed in MC3T3-E1 cells [19]. RANKL was only detected in gene expression analysis in HOBs, where its expression was twofold higher in cells incubated with a conditioned medium from MSU crystal-stimulated THP-1 monocytes than in control cells. In a previous study, an indirect effect of MSU crystals on RANKL was determined in the MLO-Y4 osteocytic cell line, where incubation with a conditioned medium from RAW264.7 cells stimulated with 0.5 mg/mL MSU crystals increased RANKL gene expression by approximately sixfold [5]. In the MLO-Y4 cells, OPG gene expression was only inhibited transiently, whereas OPG protein secretion remained unchanged. Although findings from in vitro studies and correlation analyses suggest a potential role for the disruption of RANKL/OPG balance in the development of bone erosion in gout, so far, there is no direct clinical evidence to support this role.

Imaging and pathology studies have established that tophi are closely associated with bone erosion in the gout-affected joint [3, 20]. A recent study found that the sizes of the two tophus components, the urate crystal core and surrounding inflammatory soft tissue, were independently associated with bone erosion [21]. These findings suggest that a better understanding of the inflammatory environment in and around the tophus could help identify mechanisms that underlie bone erosion in gout. In cells of the monocyte lineage used in our study, MSU crystals directly stimulated PGE_2_ and TNF-α secretion from RAW264.7 cells, and IL-1β and TNF-α secretion from THP-1 cells. Although not determined in the current study, previous studies suggest that the signaling pathways most likely to mediate the increased production of these inflammatory factors in monocyte/macrophages are NF-kappa B, C/EBP beta (CCAAT/enhancer binding protein beta), and c-Jun [22, 23]. COX-2 is also activated by IL-1α and IL-1β, a mechanism that can contribute to the increased production of PGE_2_ [24–26]. We found that MSU crystals increased IL-1β expression in THP-1 cells but not in RAW264.7 cells. IL-1α concentrations were not determined in the current study, and we cannot exclude a potential role for IL-1α in mediating the indirect effect of MSU crystals in osteoblastic cells. Secretion of PGE_2_ and TNF-α from RAW264.7 cells and human peripheral blood mononuclear cells in response to MSU crystals has been previously described [5, 27]. In vivo, characterization of the cellular component of the tophus indicated that macrophages are continuously recruited into the tophi and that mono- and multinucleated macrophages are present in the soft tissue surrounding the MSU crystal core [1, 6]. A large number of cells in the tophus express the COX-2 enzyme [5] and the inflammatory cytokines IL-1β [1] and TNF-α [6]. Bone cells are another potential source of inflammatory mediators in the gout-affected joint. In our study, a conditioned medium from MSU crystal-stimulated monocytes/ macrophages induced the expression of genes encoding COX-2 and IL-6 and the secretion of PGE_2_ and IL-6 from MC3T3-E1 cells and HOBs. A similar, indirect response to MSU crystals has been demonstrated in the osteocytic cell line MLO-Y4. In these cells, a conditioned medium from MSU crystal-stimulated RAW264.7 macrophages induced the secretion of PGE_2_, TNF-α, and IL-6 [5]. Studies of the direct response of cells of the osteoblast lineage to MSU crystals found the stimulation of PGE_2_, IL-6, and IL-8 in HOBs [14], whereas in MLO-Y4 cells, MSU had no direct effect on the expression of inflammatory genes [5]. The proinflammatory mediators that were stimulated directly in monocytic and indirectly in osteoblastic cells in our study are all known to affect both osteoclasts and osteoblasts. TNF-α, IL-6, and PGE_2_ stimulate osteoclast formation and bone resorption, particularly in pathological contexts [28–30]. PGE_2_ and IL-6 also stimulate osteoblast differentiation and bone formation [31, 32], whereas TNF-α inhibits these processes [33, 34]. Increased concentrations of the proinflammatory mediators in the extracellular environment around the tophus could contribute to the imbalanced bone remodeling that leads to bone erosion and aberrant bone formation.

We used the COX-2 selective inhibitor SC-236 to examine whether PGE_2_, which is secreted from HOBs in response to a conditioned medium from MSU crystal-stimulated THP-1 cells, acts in an autocrine fashion to induce the changes seen in HOBs. We chose to study the role of COX-2 activation because we had previously shown that the indirect effects of MSU crystals in the osteocytic cell line MLO-Y4 were reduced by COX-2 inhibition, whereas TNF-α inhibition was without effect [5]. As expected, SC-236 inhibited PGE_2_ secretion from HOBs. However, it only partially suppressed the activation of *IL6* gene expression and had no effect on IL-6 secretion, suggesting that COX-2 activation is not a central mechanism mediating this response. COX-2 activation might play a more important role in osteocytes, as a similar experiment in MLO-Y4 cells found that COX-2 inhibition strongly suppressed the induced secretion of IL-6 [5].

Our study had several limitations. We did not perform an analysis of the specific contribution of all the secreted factors identified in monocyte/ macrophage conditioned medium to the osteoblast effects. In future studies, TNF-α inhibitors, antagonists of the EP4 receptor—the main receptor that mediates PGE_2_ activity in osteoblasts [25], and the IL-1 receptor antagonist anakinra could be used to determine the role of each: TNF-α, PGE_2_, and IL-1 in MSU crystal macrophage-mediated effects in osteoblasts. The fact that this was an in vitro study could be considered another limitation relating to the clinical relevance of our findings. However, the research question addressed in this study originated from observations made in clinical samples of people with erosive gout. Specifically, histological analysis of joints affected by gout demonstrating the presence of MSU crystals, macrophage-rich tophus tissue, and areas of bone erosion provides the clinical context for our study.

## Conclusions

In summary, factors secreted from macrophages stimulated by MSU crystals attenuate osteoblast differentiation and induce the expression and secretion of proinflammatory mediators from osteoblasts. Our results suggest that disordered bone remodeling and bone erosion in joints affected by tophaceous gout result from a combination of direct and indirect effects of MSU crystals. A previous study showing that the urate crystal core and surrounding inflammatory soft tissue of the tophus are independently associated with bone erosion supports the concept of a dual, direct and indirect, effect of the tophus in bone.

## Supplementary Information

**Additional file 1:** **Figure S1.** Effects of conditioned medium from MSU crystal-stimulated THP-1 monocytes on factor secretion from HOBs. Description: The file includes a figure and a figure legend.

## Data Availability

The datasets used and analyzed during the current study are available from the corresponding author on reasonable request.
