# Supplementary material for: Factors secreted by monosodium urate crystal-stimulated macrophages promote a proinflammatory state in osteoblasts: a potential indirect mechanism of bone erosion in gout
Source: Arthritis Res Ther. 2022 Sep 5;24:212. doi: 10.1186/s13075-022-02900-z (PMC9442999; doi:10.1186/s13075-022-02900-z)
Supplement: Supplementary file 1 — Additional file 1: Figure S1. Effects of conditioned medium from MSU crystal-stimulated THP-1 monocytes on factor secretion from HOBs. Description: The file includes a figure and a figure legend. [file 13075_2022_2900_MOESM1_ESM.pdf]

Figure S1

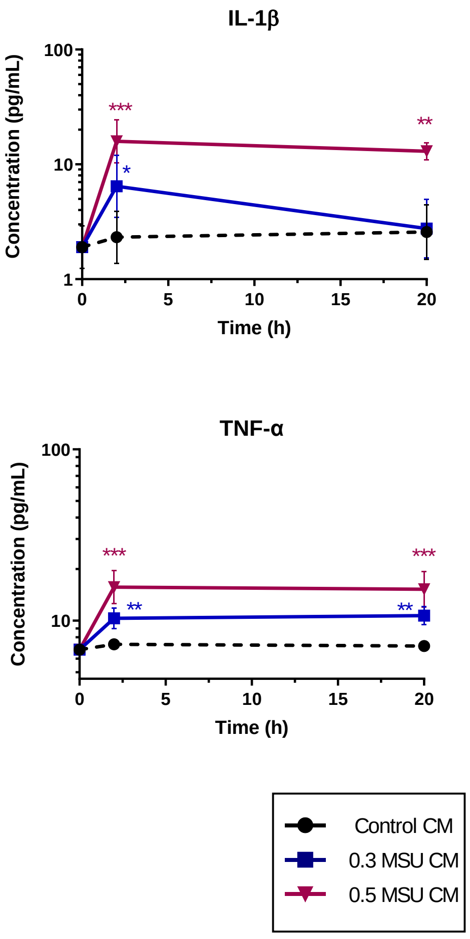

Figure S1 Effects of conditioned medium from MSU crystal-stimulated THP-1 monocytes on factor secretion from HOBs THP-1 monocytes were cultured with MSU crystals for 20 h. Conditioned media from THP-1 cultures were added to HOB cells at 40% of the final volume. The concentrations of IL-1β and TNF-α secreted from HOBs was measured by ELISA. Means (SEM) of data pooled from three or more biological repeats are presented. Data were analyzed by two-way ANOVA with post-hoc Dunnett's test. $^*p < 0.05$, $^{**}p < 0.01$, and $^{***}p < 0.001$ versus control conditioned medium at that time point. MSU CM, concentrations of MSU used to prepare conditioned medium from THP-1 cells.
